# Inhibition of Hb Binding to GP1bα Abrogates Hb-Mediated Thrombus Formation on Immobilized VWF and Collagen under Physiological Shear Stress

**DOI:** 10.1371/journal.pone.0154276

**Published:** 2016-04-22

**Authors:** Gowtham K. Annarapu, Rashi Singhal, Yuandong Peng, Prasenjit Guchhait

**Affiliations:** 1 Disease Biology Laboratory, Regional Centre for Biotechnology, National Capital Region Biotech Science Cluster, Faridabad, India; 2 Biotechnology Department, Manipal University, Karnataka, India; 3 Cardiovascular Research Section, Department of Medicine, Baylor College of Medicine, Houston, Texas, United States of America; Monash University, AUSTRALIA

## Abstract

**Background:**

Reports including our own describe that intravascular hemolysis increases the risk of thrombosis in hemolytic disorders. Our recent study shows that plasma Hb concentrations correlate directly with platelet activation in patients with paroxysmal nocturnal hemoglobinuria (PNH). The binding of Hb to glycoprotein1bα (GP1bα) increases platelet activation. A peptide AA1-50, designed from N-terminal amino acid sequence of GP1bα significantly inhibits the Hb binding to GP1bα as well as Hb-induced platelet activation. This study further examined if the Hb-mediated platelet activation plays any significant role in thrombus formation on subendothelium matrix under physiological flow shear stresses and the inhibition of Hb-platelet interaction can abrogate the above effects of Hb.

**Methods and Results:**

Study performed thrombus formation assay *in vitro* by perfusing whole blood over immobilized VWF or collagen type I in presence of Hb under shear stresses simulating arterial or venous flow. The Hb concentrations ranging from 5 to 10 μM, commonly observed level in plasma of the hemolytic patients including PNH, dose-dependently increased thrombus formation on immobilized VWF under higher shear stress of 25 dyne/cm^2^, but not at 5 dyne/cm^2^. The above Hb concentrations also increased thrombus formation on immobilized collagen under both shear stresses of 5 and 25 dyne/cm^2^. The peptide AA1-50 abrogated invariably the above effects of Hb on thrombus formation.

**Conclusions and Significance:**

This study therefore indicates that the Hb-induced platelet activation plays a crucial role in thrombus formation on immobilized VWF or collagen under physiological flow shear stresses. Thus suggesting a probable role of this mechanism in facilitating thrombosis under hemolytic conditions.

## Introduction

Intravascular hemolysis increases the risk of thrombosis in hemolytic disorders. Studies including our own describe a significant association between the elevated plasma Hb and vascular dysfunction and prothrombotic events in hemolytic disorders including PNH [1–4]. The direct correlation of plasma Hb with the occurrence and severity of intravascular thrombosis in PNH is reported, wherein venous [5,6] and arterial [7,8] thrombosis are principal causes of morbidity and mortality in these patients. Studies have also suggested that the activation of platelets, most likely mediated via the scavenging of nitric oxide (NO) by Hb [9,10] is one of the potential causes of thrombosis in PNH [11,12]. It has been shown that NO inhibits platelet aggregation, induces disaggregation of aggregated platelets, and inhibits platelet adhesion via the cyclic guanosine monophosphate (GMP) pathway [13]. Furthermore, our recent study shows that Hb can bind directly to GP1bα on platelet surface and induce its activation, and this activation is diminished in the presence of a peptide AA1-50 (designed from N-terminal domain of GP1bα, M1-T50) that blocks Hb binding to platelet [1]. To develop more insight into the prothrombotic role of extracellular Hb this study further examined if the Hb concentrations, commonly observed levels in hemolytic patients including PNH [1–4] can induce thrombus formation on subendothelium matrix under physiological flow shear stresses *in vitro*; and if the inhibition of Hb-platelet interaction can abrogate the above effects of Hb.

## Materials and Methods

The synthetic peptides AA1-50 (mpllllllllpsplhphpicwvskvashlevncdkrnltalppdlpkdtt) designed from N-terminal domain of GP1bα) and scrambled control peptide (msplekcplstvcldtplnhkvlpelpvlilplthnalradkplsdlplh) used in our recent work [1] were purchased from GL BioChem, Shanghai. Majority of other laboratory chemicals including normal adult Hb (HbA with a purity of 98.5% isolated through Sephadex G-25 column) were purchased from Sigma-Aldrich, St. Louis, USA.

### Blood sample

To collect blood samples, approval was obtained from the Institutional Ethics Committee for Human Research of the Regional Centre for Biotechnology. Informed consent was provided according to the recommendations of the declaration of Helsinki. Normal healthy volunteers (n = 9) were recruited following written consent and 25–30 mL of blood sample was collected in 0.32% sodium citrate anticoagulant.

### Parallel flow chamber: Platelet thrombus formation assay

The whole blood collected from healthy individuals was perfused over the petri plate coated with VWF (purified from plasma) or collagen (type-1, Sigma, St. Louis, USA). The purification of plasma VWF is mentioned in our earlier work [14]. Whole blood was incubated for 15 min with different concentrations of Hb before perfusion. A syringe pump (Harvard Apparatus Inc., USA) was connected to the outlet port that drew blood through the chamber at different shear stresses 5 or 25 dyne/cm^2^. The flow chamber was mounted onto a Nikon Eclipse Ti-E inverted stage microscope (Nikon, Japan) equipped with a high-speed digital camera. Movie was recorded at magnification 40X and analyzed using NIS-Elements version 4.2 software.

### Statistical analysis

Experimental values were presented as mean ± standard error of mean (SEM). The Student’s t-test (paired) and two-way ANOVA was used for data analysis, and a p-value less than 0.05 was considered to be statistically significant.

## Results

### Peptide AA1-50 abrogates Hb-mediated thrombus formation on immobilized VWF under arterial shear stresses

**Fig 1:**
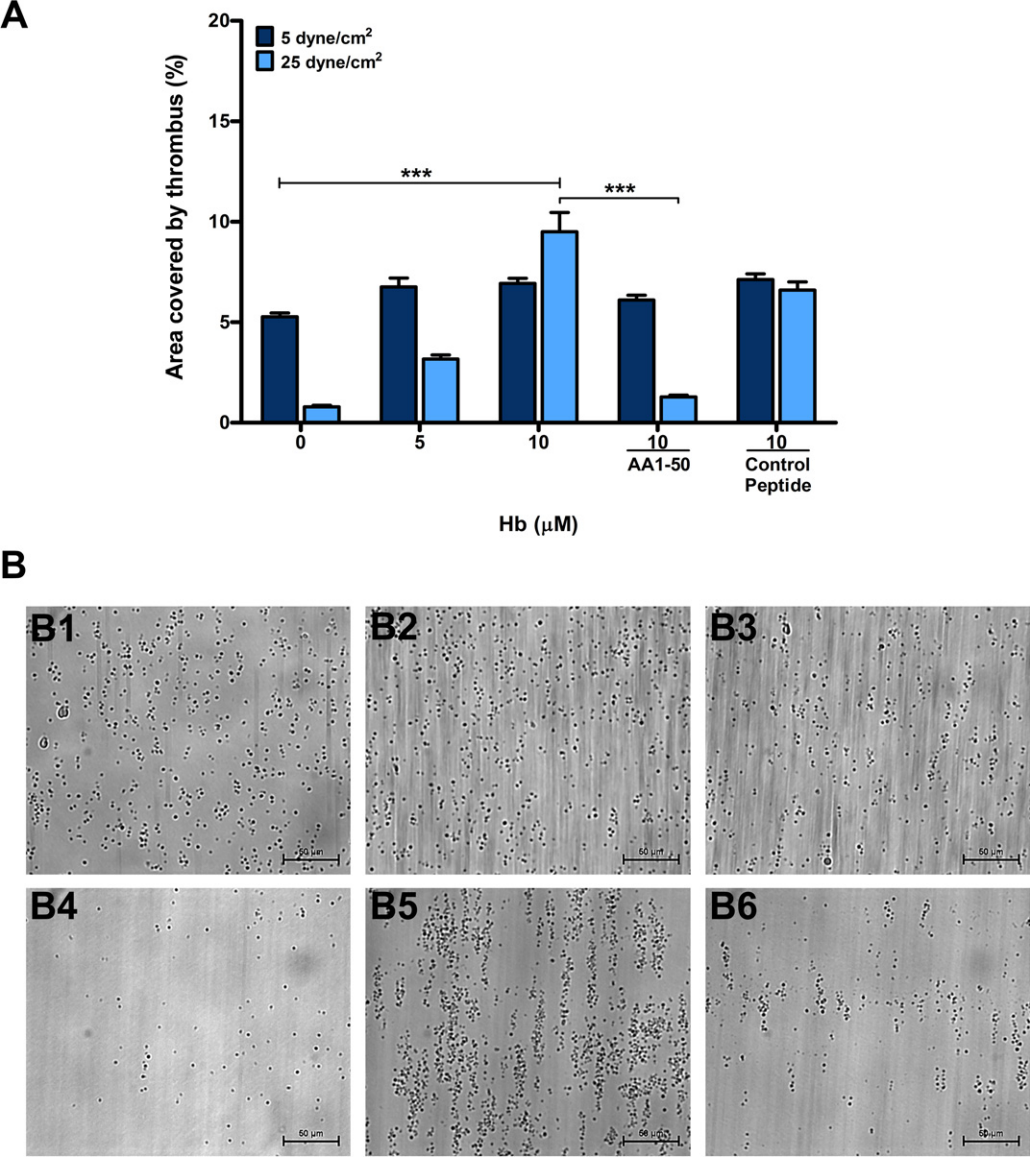
Platelet thrombus formation on VWF surface in the presence of Hb. **(A)** Whole blood was perfused over immobilized VWF (100 μg/mL) under the shear stresses of 5 and 25 dyne/cm^2^ in presence of Hb (μM). Data are the mean ± SEM of the area covered by thrombus on VWF surface as calculated from ten fields of view from three independent experiments. The Hb increased the thrombus area in a concentration-dependent manner under 25 dyne/cm^2^, ***P < 0.0001, the size of thrombus was further abrogated by peptide AA1-50 (5 μM), ***P < 0.0001, but not by the control peptide. **(B)** The 40X images show that platelet thrombus was increased in presence of Hb (10 μM) under shear of 25 dyne/cm^2^ **(B5)** when compared with no-Hb **(B4)**, and the thrombus was further decreased by the AA1-50 (5 μM) **(B6)**. Whereas under 5 dyne/cm^2^, the platelet thrombus did not show any significant change in presence of Hb (10 μM) **(B2)** when compared with no-Hb **(B1)**. Platelet thrombus in presence of Hb (10 μM) and AA1-50 (5 μM) **(B3)**.

The Hb concentrations ranging from 5 to 10 μM, commonly observed level in plasma of the hemolytic patients, increased the formation of platelet thrombus in a concentration-dependent manner when perfused with whole blood over immobilized VWF (100 μg/mL, purified from plasma of normal individual) under shear stress of 25 dyne/cm^2^ simulating arterial blood flow (Fig 1A, 1B4 and 1B5). The synthetic peptide AA1-50, designed from N-terminal domain of GP1bα (M1-T50), which blocks Hb binding to platelet [1] also inhibited significantly the above effects of Hb (Fig 1A and 1B6). Unlike in higher shear, Hb did not show any significant effects on thrombus formation when the above experiment was performed under a venous blood flow shear stress of 5 dyne/cm^2^, (Fig 1A, 1B1 and 1B2).

### Peptide AA1-50 also inhibits thrombus formation on immobilized collagen under both venous and arterial shear stresses

**Fig 2:**
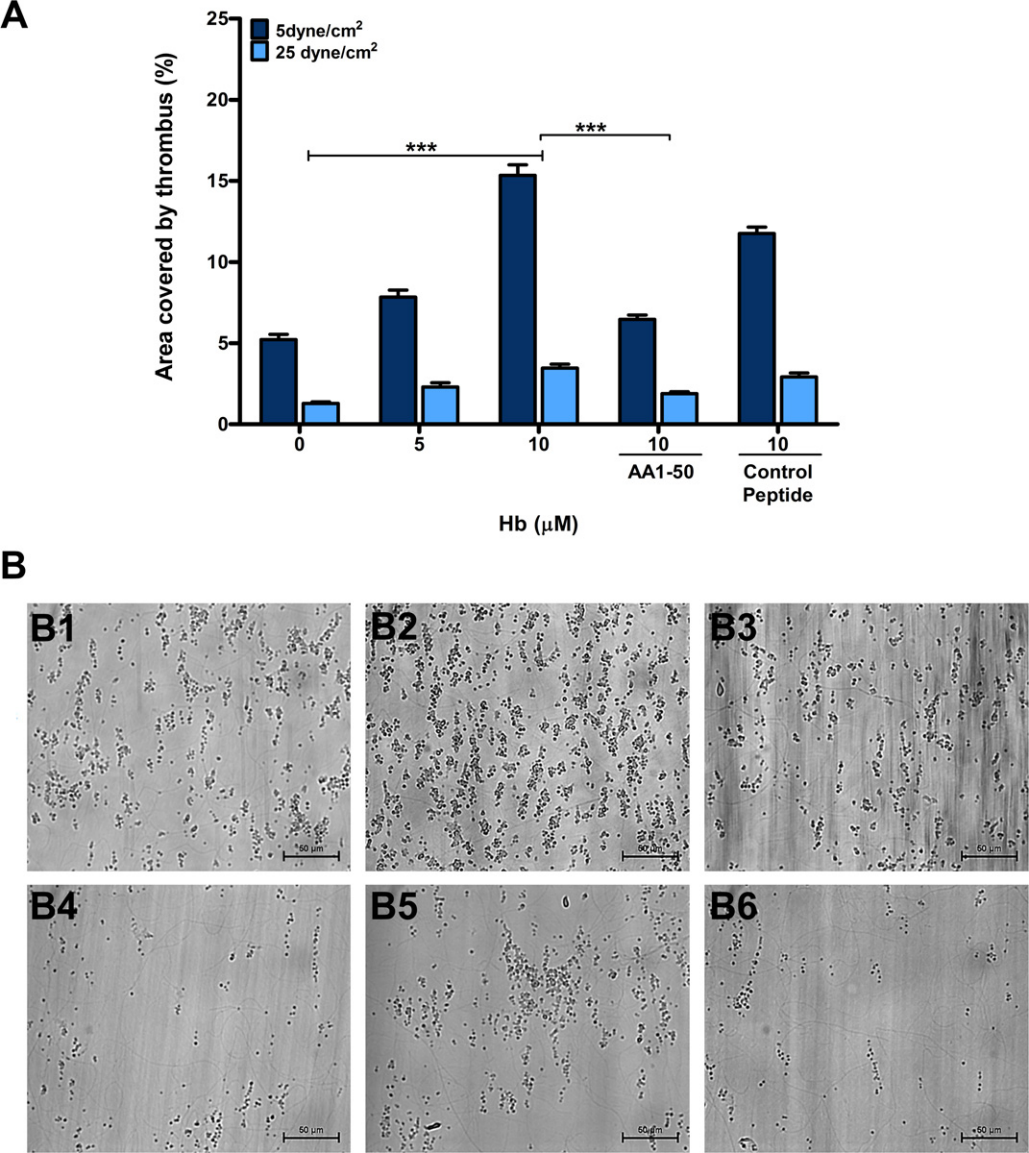
Platelet thrombus formation on collagen surface in the presence of Hb. **(A)** Whole blood was perfused over immobilized collagen type-1 (100 μg/mL) under shear stresses of 5 and 25 dyne/cm^2^ in the presence of Hb. Data are the mean ± SEM of the area covered by thrombus on collagen surface from three independent experiments. The Hb increased the thrombus formation in concentration-dependent manner for both shear stresses, ***P < 0.0001, which was further inhibited by the peptide AA1-50 (5 μM), ***P < 0.0001. **(B)** The 40X images show that thrombus size was increased in presence of Hb (10 μM) under 5 dyne/cm^2^ **(B2)** when compared with no-Hb **(B1)**, and thrombus was further decreased by the peptide AA1-50 (5 μM) **(B3)**. Similarly under shear stress of 25 dyne/cm^2^, the thrombus area was increased in presence of Hb **(**10 μM) **(B5)** when compared with no-Hb **(B4),** and thrombus was further decreased by the peptide AA1-50 (5 μM) **(B6)**.

Furthermore under the above experimental conditions, Hb also increased the platelet thrombus formation over immobilized collagen type-1 (100 μg/mL) at both shear stresses of 5 dyne/cm^2^ (Fig 2A, 2B1 and 2B2) and 25 dyne/cm^2^ (Fig 2A, 2B4 and 2B5) in a concentration-dependent manner, although the size of thrombus was observed larger in case of lower shear stress compared to higher shear. The peptide AA1-50 significantly abrogated the above effects of Hb on thrombus formation under both shear stresses (Fig 2A, 2B3 and 2B6).

## Discussion

We have recently described that Hb binding to GP1bα increases platelet activation [1]. We further show in this study that Hb concentrations ranging from 5 to 10 μM, commonly observed level in plasma of PNH patients [1] significantly increases thrombus formation *in vitro* on immobilized VWF under arterial shear stress (Fig 1A, 1B4 and 1B5) but not under lower shear stress such as 5 dyne/cm^2^ that simulate venous blood flow (Fig 1A, 1B1–1B3). More importantly, the peptide AA1-50, which inhibits Hb binding to GP1bα as well as Hb-induced activation of platelets [1], also blocks the Hb-mediated thrombus formation on immobilized VWF (Fig 1A and 1B6). This suggests that Hb plays a crucial role in activating and adhering platelets to VWF surface under high shear stresses. In circulating blood, initial platelet adhesion to the injured vessel wall is mediated by interaction between GP1bα on platelet surface and A1 domain of VWF [15], and this interaction is crucially modulated by the hydrodynamic high shear stress and/or immobilization of the VWF multimers [16,17].

We further show that the Hb (5 and 10 μM) also promotes platelet thrombus formation on immobilized collagen type 1 in a concentration-dependent manner under both shear stresses of 5 dyne/cm^2^ (Fig 2A, 2B1 and 2B2) and 25 dyne/cm^2^ (Fig 2A, 2B4 and 2B5). The platelet thrombus formation is significantly inhibited by the peptide AA1-50, which blocks the Hb-mediated platelet activation (Fig 2A, 2B3 and 2B6). This study therefore indicates that the Hb-mediated platelet activation plays a very crucial role in thrombus formation on immobilized VWF or collagen under physiological flow shear stresses. Thus suggesting a probable role of this mechanism in facilitating thrombosis under hemolytic conditions.
